# Changing epidemiology and challenges of malaria in China towards elimination

**DOI:** 10.1186/s12936-019-2736-8

**Published:** 2019-03-29

**Authors:** Shengjie Lai, Junling Sun, Nick W. Ruktanonchai, Sheng Zhou, Jianxing Yu, Isobel Routledge, Liping Wang, Yaming Zheng, Andrew J. Tatem, Zhongjie Li

**Affiliations:** 1 WorldPop, School of Geography and Environmental Science, University of Southampton, Southampton, UK; 2 Division of Infectious Disease, Key Laboratory of Surveillance and Early–Warning on Infectious Disease, Chinese Center for Disease Control and Prevention, Beijing, China; 3 School of Public Health, Fudan University, Key Laboratory of Public Health Safety, Ministry of Education, Shanghai, China; 4 Flowminder Foundation, Stockholm, Sweden; 5 MOH Key Laboratory of Systems Biology of Pathogens and Christophe Mérieux Laboratory, CAMS-Fondation Mérieux, Institute of Pathogen Biology, Chinese Academy of Medical Sciences & Peking Union Medical College, Beijing, China; 6 Department of Infectious Disease Epidemiology, Imperial College London, London, UK

**Keywords:** Malaria, Epidemiology, Elimination, Importation, China, Africa, Southeast Asia

## Abstract

**Background:**

Historically, malaria had been a widespread disease in China. A national plan was launched in China in 2010, aiming to eliminate malaria by 2020. In 2017, no indigenous cases of malaria were detected in China for the first time. To provide evidence for precise surveillance and response to achieve elimination goal, a comprehensive study is needed to determine the changing epidemiology of malaria and the challenges towards elimination.

**Methods:**

Using malaria surveillance data from 2011 to 2016, an integrated series of analyses was conducted to elucidate the changing epidemiological features of autochthonous and imported malaria, and the spatiotemporal patterns of malaria importation from endemic countries.

**Results:**

From 2011 to 2016, a total of 21,062 malaria cases with 138 deaths were reported, including 91% were imported and 9% were autochthonous. The geographic distribution of local transmission have shrunk dramatically, but there were still more than 10 counties reporting autochthonous cases in 2013–2016, particularly in counties bordering with countries in South-East Asia. The importation from 68 origins countries had an increasing annual trend from Africa but decreasing importation from Southeast Asia. Four distinct communities have been identified in the importation networks with the destinations in China varied by origin and species.

**Conclusions:**

China is on the verge of malaria elimination, but the residual transmission in border regions and the threats of importation from Africa and Southeast Asia are the key challenges to achieve and maintain malaria elimination. Efforts from China are also needed to help malaria control in origin countries and reduce the risk of introduced transmission.

**Electronic supplementary material:**

The online version of this article (10.1186/s12936-019-2736-8) contains supplementary material, which is available to authorized users.

## Background

*Plasmodium* malaria, transmitted via the bites of female *Anopheles* mosquitoes, is one of the most prevalent parasitic diseases affecting mankind. Although the global malaria burden has fallen from an estimated 239 million cases occurred worldwide in 2010 to 219 million cases in 2017, no significant progress in reducing global malaria cases was made for the first time in the last decade, especially between 2015 and 2017 [1–4]. However, the progress of eliminating malaria in China seems to be encouraging.

Malaria was once widespread in China, with more than 90% population in China were estimated at risk of infection in the 1940s, and it was still highly endemic in China between 1950s and 1970s, with the highest record of 24 million cases reported in 1970 [5, 6]. Due to the widely use of anti-malarial medications, along with the unprecedented socioeconomic changes and urbanization in China, the incidence of malaria decreased gradually from 1980 to 2000, with only 20 cases per one million residents in 2000 [5, 6]. Although the resurgence of malaria occurred in central China between 2001 and 2006 [7, 8], the efforts of intensified control since 2007 resulted in a dip in the number of cases, reducing to less than 6 cases per one million residents in 2010 [9]. Subsequently, a National Malaria Elimination Programme (NMEP) was launched in China in May 2010, aiming at achieving malaria elimination by 2020 [5, 10], and comprehensive intervention strategies have been adopted, e.g. the “1-3-7” approach: reporting cases within 1 day, investigation within 3 days, and response to prevent further transmission within 7 days [11, 12]. Consequently, the geographic range of locally transmitted malaria has shrunk dramatically in China, and for the first time zero indigenous cases were detected in 2017 [4, 13]. With the rapid growth of Chinese oversea travel and increasing investment in overseas projects from China in the last decade, however, imported malaria has been increasingly reported in China [14–17], and the introduced parasites might cause the resurgence of local transmission, which has occurred in countries that have eliminated malaria [18, 19].

In light of the historical achievement of eliminating malaria in China, a comprehensive analysis is needed to elucidate the changing epidemiology and challenges of malaria faced by China towards nationwide malaria elimination. Based on a contemporary dataset of individual cases, here an integrated analysis was conducted to determine the spatiotemporal and demographic features of autochthonous and imported malaria, the areas of residual local transmission, and the origin–destination networks of malaria importation. The evidence of this work helps to identify the challenges of eliminating malaria in China and support the update of precise strategies for NMEP and future needs in post-elimination era.

## Methods

### Data sources

The data of individual malaria cases were obtained from the Malaria Enhanced Surveillance Information System (MESIS) in China. The MESIS was launched in 2010 to actively collect epidemiological information of malaria cases (Additional file 1: Table S1), required by the Technical Scheme of China Malaria Elimination [11, 20]. All clinically diagnosed and laboratory-confirmed individual malaria cases reported in MESIS during 2011–2016 were included in this study. Laboratory-confirmed malaria cases referred to patients with a positive result from one of the laboratory tests including rapid diagnostic tests (RDTs), microscopy, or polymerase chain reaction (PCR): RDTs were the primary diagnostic tools in local level; microscopy was the gold standard method for case verification used in county, prefectural and provincial levels; and as PCR was more sensitive than microscopy, it was mainly used as a confirmatory technique for laboratory diagnosis of malaria at provincial levels for cases who had no parasites detected in blood examination or for identifying malaria parasites at the species level when microscopy was equivocal [21, 22]. Clinically diagnosed cases, including species classification of these cases, were diagnosed according to symptoms, response to therapy, any previous diagnosis of malaria, and predominate species in the geographic origin of infection. An imported case was a malaria case who travelled to any malaria-endemic areas outside China within the month before illness onset, and the last country visited with ongoing malaria transmission was taken as the potential location of infection [23]. Otherwise, a malaria case was considered to be a probable autochthonous case [11].

### Data analysis

The epidemiologic characteristics of malaria cases were summarized by autochthonous and imported malaria. The annual malaria incidence rate was calculated for imported, local, and all cases, respectively, by using the corresponding population data obtained from the National Statistical Bureau of China [24]. The annual incidence rate by county was mapped to define and compare the geographic distributions of autochthonous and imported malaria. Moreover, to present the space-explicit distribution of malaria in China, a smoothly tapered density surface based on the coordinates of individual cases was fitted by using the quartic kernel function and bandwidth described by Silverman [25]. The output density surface was compiled at a spatial resolution of 0.083333 decimal degrees per pixel (approx. 10 km at the Equator). The gridded geographic distributions of dominant *Anopheles* vectors, estimated by the Malaria Atlas Project using the boosted regression tree modelling approach [26], were overlaid with the polygons of counties with locally transmitted malaria in 2011–2016 to explore the potential high risk areas of residual transmission in China.

Based on the travel history of cases, the potential origin–destination routes of malaria importation from other endemic countries into 31 provinces of mainland China were identified [27]. To quantify the connectivity of malaria importation, the origin–destination networks were divided into communities densely connecting the origins (endemic countries) and the destinations (provinces in China) by modularity analysis, which compared the number of links inside a given module with the expected value for a randomized graph of the same size and same degree sequence [27–29]. Networks with high modularity have dense connections between the nodes within communities but sparse connections between nodes in different communities. The number of communities of networks is defined by the modularity optimization process, but it can be changed by setting resolution parameter (between 0.1 and 2), and a lower value can generate more communities [30, 31]. As the default value (1) of resolution parameter in modularity analysis only detected three communities with the largest group containing more than 90% origins and destinations of the importation networks, we used a resolution value of 0.9 to generate more small communities to understand the connectivity of this importation phenomenon.

Moreover, a linear regression model and a nonlinear regression model with cosine function [32] were used to explore the annual and seasonal trends of *Plasmodium* malaria importation by origins (Africa and Southeast Asia) and species (*Plasmodium vivax* and *Plasmodium falciparum*). A scatterplot was plotted to explore the correlation between malaria importation and autochthonous transmission in origin countries. The data of presumed and confirmed malaria cases reported by country in 2011–2016 were extracted from the annual World Malaria Report in 2017 published by the World Health Organization [1], and the corresponding population data published by United Nations [33] were used to define the average incidence rate of malaria reported by country. The *R* statistical software (Version 3.3.1, R Foundation for Statistical Computing, Vienna, Austria) and ArcGIS 10.3 (ESRI, Redlands, CA, USA) were used to data collation and analyses, and the Gephi 0.91 software was used to detect the communities of importation networks [34].

## Results

### Autochthonous malaria

**Fig. 1:**
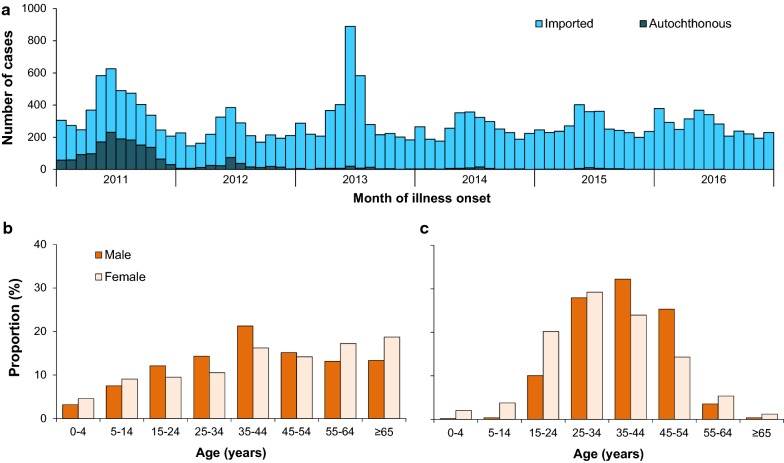
Epidemic curve and demographic features of malaria in China. **a** Epidemic curve of autochthonous and imported malaria (n = 21,062) in China, 2011–2016. **b** Age of autochthonous male (n = 1223) and female cases (n = 685) in 2011–2016. **c** Age of imported male (n = 18,069) and female cases (n = 1085) in 2011–2016

**Fig. 2:**
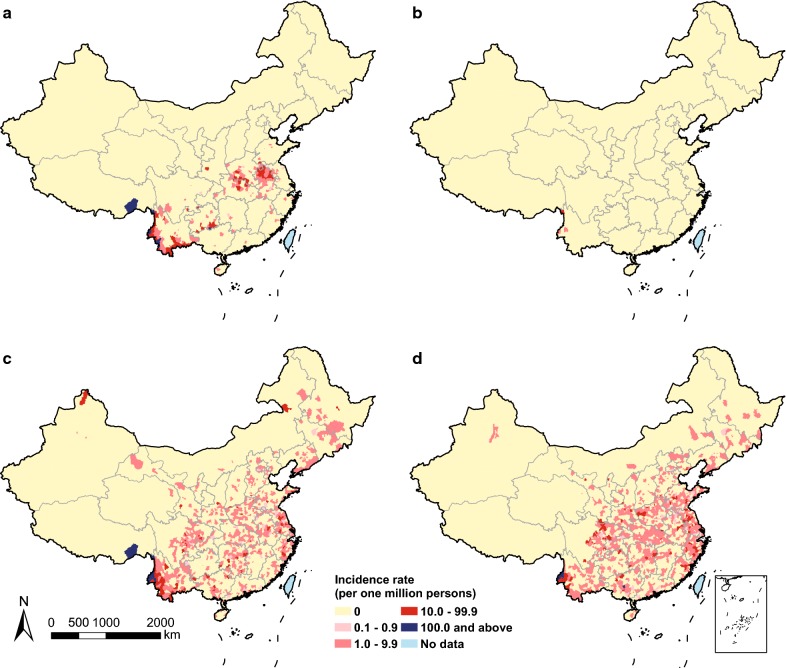
Geographic distribution of autochthonous and imported malaria by county in China, 2011 and 2016. Incidence rate of autochthonous malaria in **a** 2011 and **b** 2016. Incidence rate of imported malaria in **c** 2011 and **d** 2016

A total of 21,062 malaria cases were reported between 2011 and 2016, of which 91% were imported and 9% were autochthonous (Additional file 2: Table S2). The number of autochthonous cases dropped from 1469 cases (1.1 cases per one million persons) in 2011 to 3 cases (0.002) in 2016 (Fig. 1a). Among autochthonous cases, most (90%) were infected with *P. vivax*, while *P. falciparum* was detected in most imported cases (62%). With the rapid shrinking geographic distribution of autochthonous malaria transmission, all counties in the border areas of Yunnan have reduced annual incidence rate to less than one case per 10,000 persons since 2013, and most counties have reduced autochthonous case to zero in 2016 (Fig. 2 and Additional file 3: Fig. S1). Only three autochthonous cases have been detected in counties along the border between Myanmar and Yunnan province in 2016.

**Fig. 3:**
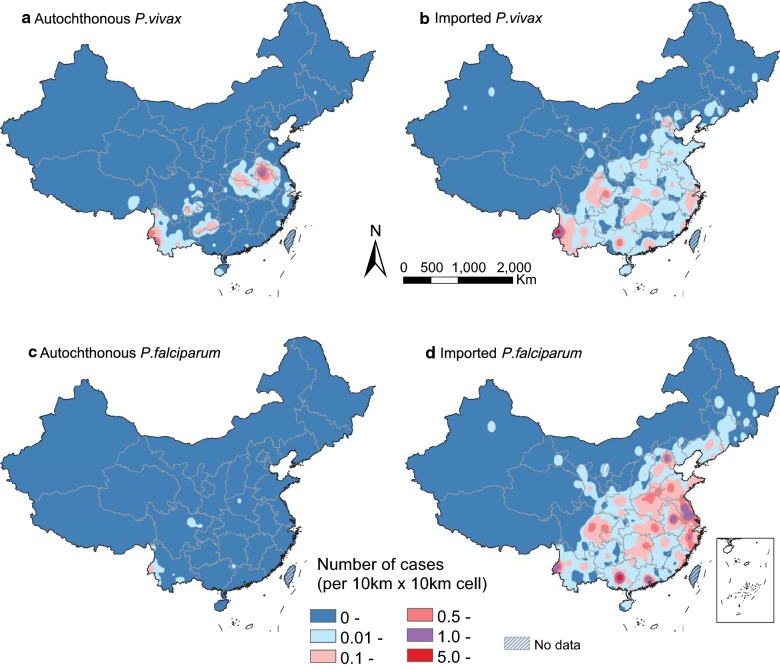
Kernel density of autochthonous and imported malaria cases by species, 2011–2016. **a** Autochthonous *P. vivax*. **b** Imported Autochthonous *P. vivax*. **c** Autochthonous *P. falciparum*. **d** Imported *P. falciparum*

Among the counties with only *Anopheles sinensis* and/or *Anopheles lesteri* as dominant vectors, the number of *P. vivax* and *P. falciparum* cases have been reduced quickly in 2011–2013 (Additional file 4: Fig. S2). However, among the counties with other dominant vectors (e.g. *Anopheles minimus* sensu lato. *Anopheles dirus sl*, *Anopheles stephensi*, and *Anopheles maculatus*), there were still more than 10 counties reporting autochthonous cases in 2013–2016, particularly in counties of Yunnan province bordering with Myanmar and Laos, the southeast region of Tibet bordering with India, and Hainan island in the South China Sea (Fig. 3 and Additional file 3: Fig. S1). Additionally, 138 deaths were found in our dataset, and the case fatality risk of imported cases (0.7%) was higher than that (0.1%) of autochthonous cases. All 137 deaths of imported cases were detected in the counties without local malaria transmission in 2011–2016, with most deaths in imported cases (94.2%, 129/137) and the only death in autochthonous cases were infected with *P. falciparum*.

### Imported malaria

**Fig. 4:**
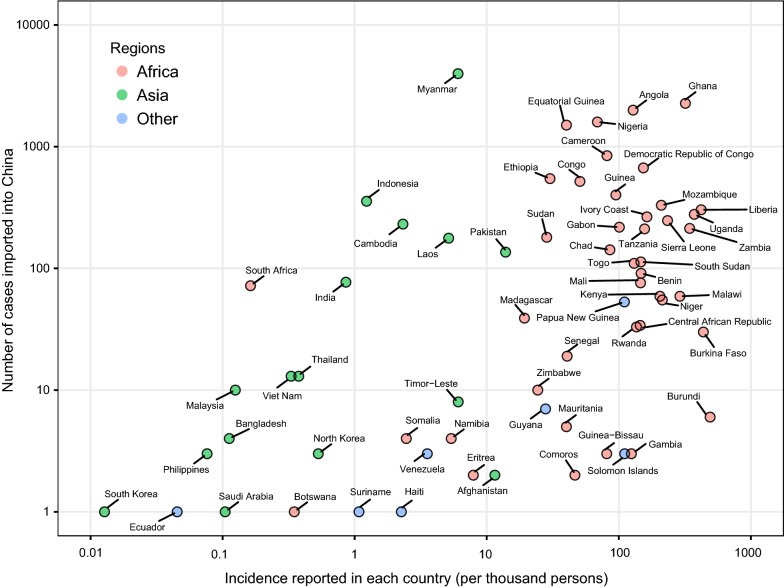
The number of imported malaria cases reported in China and the incidence of malaria reported in each origin country, 2011–2016. The y-axis shows the log of number of malaria cases imported from different countries into China. The x-axis shows log of the annual average incidence rate reported in each country. This figure includes 65 countries with available incidence data reported in the World Malaria Report in 2017 [1]. The colours of points show the continents in where the countries are located

**Fig. 5:**
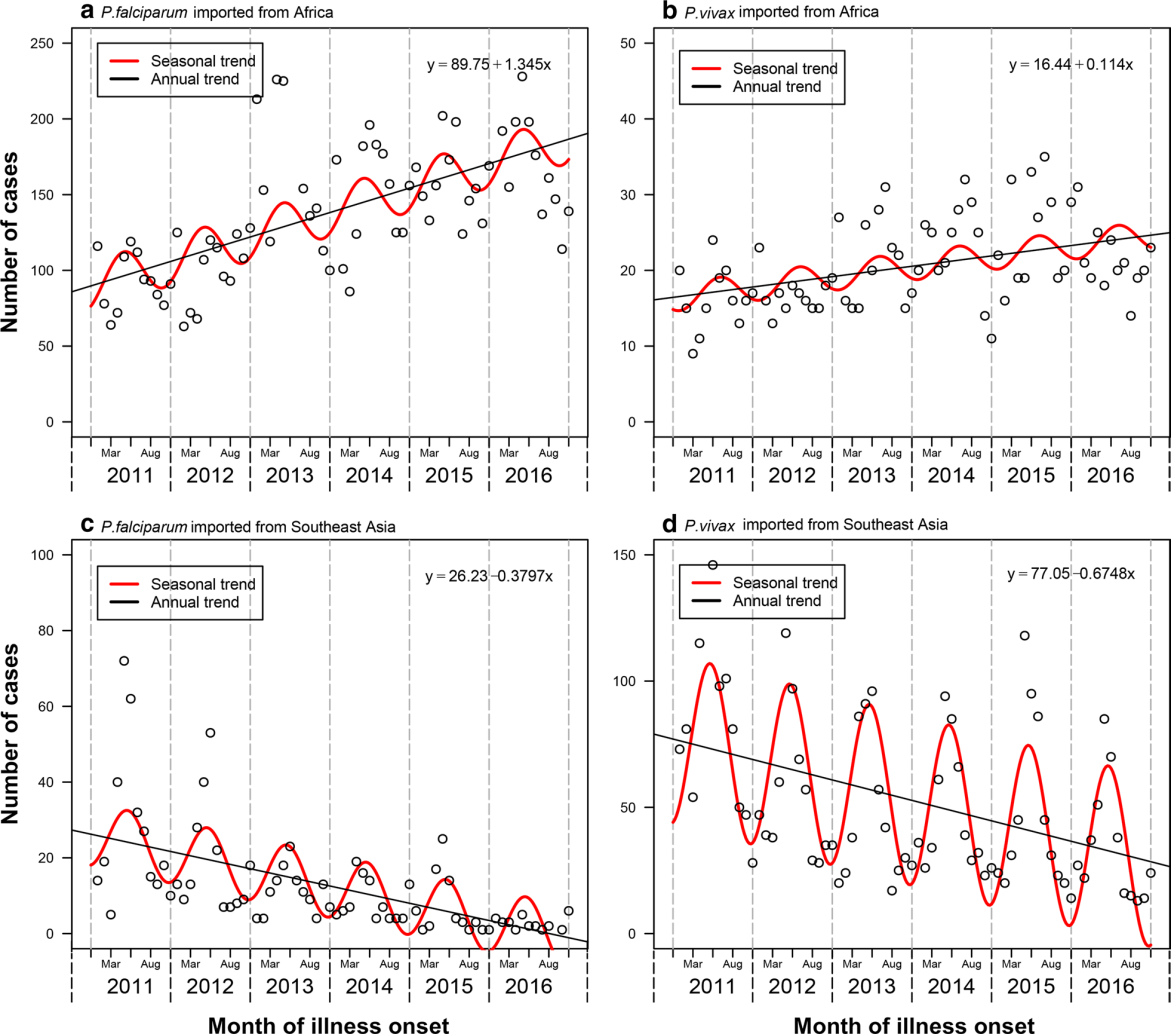
Trend of imported Plasmodium malaria cases by origins and species, 2011–2016. **a** *P. falciparum* imported from Africa. **b** *P. vivax* imported from Africa. **c** *P. falciparum* imported from Southeast Asia. **d** *P. vivax* imported from Southeast Asia. The seasonality of imported cases was fitted by nonlinear regression with cosine function proposed in a previous study [32], and a linear regression model for the annual trend was fit with the equations provided

A total of 19,154 cases imported from 68 countries were reported in mainland China during 2011–2016, with most cases from Africa (72%) and Southeast Asia (25%). The magnitude of importations might be partially related to the endemicity of malaria in origins (Fig. 4). Most cases (79%) imported from Africa (n = 13,728) were infected with *P. falciparum*, while *P. vivax* was detected in 79% cases from Southeast Asia (n = 4791) (Additional file 5: Table S3). Both *P. falciparum* and *P. vivax* imported from Africa had an increasing annual trend and seasonal fluctuations during 2011–2016 (Fig. 5). However, the rate of cases imported from Southeast Asia showed an annual downwards trend with a wide seasonal amplitude of monthly counts. Moreover, compared with autochthonous cases, a strong male predominance (17:1 vs 2:1) and a higher proportion (95% vs 58%) of population aged 15–45 years were found in imported cases (Fig. 1b, c). Most imported cases were Chinese (94%) and migrant workers (80%), and Chinese cases had a longer stay (median 324 days; interquartile range [IQR] 168–547) in Africa than cases from South and Southeast Asia (120 days; 59–240).

**Fig. 6:**
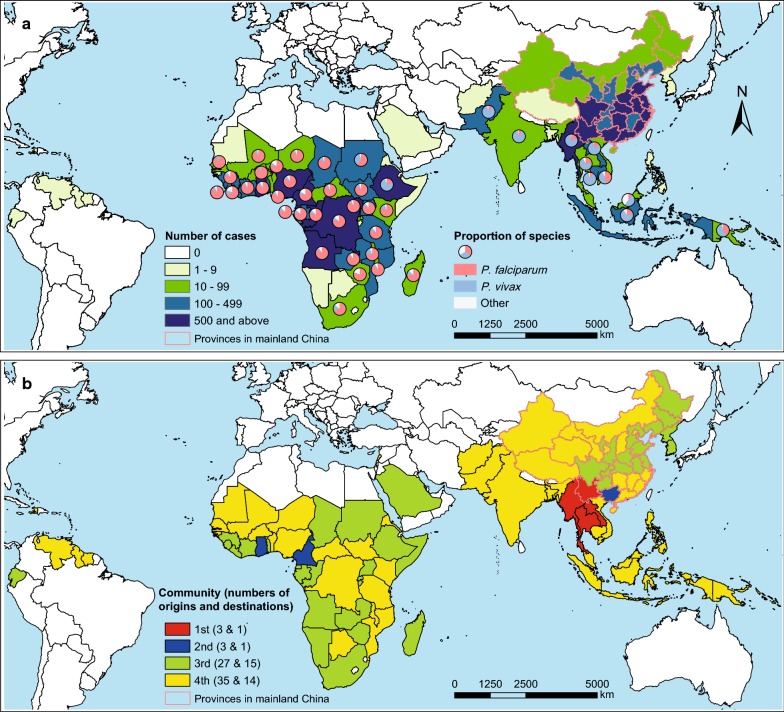
Origin-destination networks and communities of malaria importation into China, 2011–2016. **a** The numbers and species of malaria imported from origins (68 countries) into destinations (31 provinces) in mainland China. **b** Four communities of the malaria importation networks. The proportion of *Plasmodium* species are presented for countries with ≥ 10 cases

There were 921 origin–destination routes of malaria importation from malaria endemic countries into provinces in mainland China, with a median of four imported cases (IQR 1–12) of each route in 2011–2016. Myanmar (21% cases), Ghana (12%), and Angola (10%) were top three origins, and Yunnan (19%), Guangxi (12%), and Jiangsu (10%) provinces in China ranked as top three destinations (Fig. 6a). Four distinct communities have been identified by the modularity analysis (Fig. 6b and Additional file 6: Table S4). The first community included Yunnan Province and three neighbouring countries (Myanmar, Laos, and Thailand), and this community had the highest number of malaria cases imported from Southeast Asia (3406 cases), especially from Myanmar (3279 cases). The second community included Guangxi province and three African countries (Ghana, Cameroon, and Comoros) with Ghana-Guangxi link had the highest number of cases (1633) from Africa. The third community contained provinces in central and north-eastern China and neighbouring countries in East Asia and remote origin in Africa. The fourth community was constituted of provinces in north-western and southern China, neighbouring countries in South and Southeast Asia, and origin countries in western and central Africa, and Latin America.

Moreover, the geographic range of imported cases in China varied by origin and species (Fig. 3 and Additional file 7: Fig. S3): Yunnan province mainly imported malaria from Southeast Asia, in particular *P. vivax* from Myanmar; while Guangxi province in southern China, provinces in eastern China, and Sichuan in western China, and metropolitans, i.e. Beijing, Shanghai, Guangzhou with international airport hubs had imported most malaria from Africa, especially for *P. falciparum*. The co-occurrence of autochthonous and imported malaria was mainly found in the border areas of Yunnan with malaria imported from Southeast Asia, and the provinces of central China with parasites imported from Africa (Fig. 2 and Additional file 4: Fig. S1).

## Discussion

This study presents the changes in the epidemiological characteristics of autochthonous and imported malaria for China at the malaria elimination stage, and we found that malaria is on the verge of reaching the goal of elimination in China by 2020. The shrinking maps of autochthonous malaria since 2010 could partly be attributed to robust surveillance systems for rapidly detecting individual cases and response by the ‘1-3-7’ strategy rolled out nationally [12]. The success might also be contributed by improved housing of the unprecedented urbanization in China and the extensive use of residual insecticides, which reduce contact with this mosquito and vectorial capacity [35, 36]. For instance, high coverage (> 90%) of long-lasting insecticidal nets and indoor residual spraying has been achieved in southern China along the Myanmar border since 2010 [37]. Moreover, as China has an active role in the Greater Mekong Subregion (GMS) for eliminating malaria in all GMS countries by 2030 [38], the progress in China is being a major contributor towards the ambition of global malaria eradication [39].

However, the residual malaria transmission in border areas, together with the importation of parasites, has posed a great threat to successfully eliminate malaria in China. The residual local transmission of *P. vivax* and *P. falciparum* malaria during the elimination stage might reflect the spatial variability and complexity of *Anopheles* vectors in China. For instance, the *Anopheles minimus* sensu lato. and *Anopheles dirus sl*, which may have higher capacity to transmit malaria, are the dominant vectors in Yunnan province, southern parts of Tibet, and Hainan Island [26]. Moreover, climate plays an important role in vector distribution and capacity of malaria transmission [40–42]. A warming climate may potentially expand the environmentally suitable area for malaria vectors, leading to malaria resurgence [43]. For example, the resurgence of malaria occurred in central China between 2001 and 2006, which might be attributed to the increasing vectorial capacity and the basic reproductive rate of *An. sinensis* caused by the changing meteorological factors [7, 8]. Therefore, strategies to achieve and maintain malaria elimination in China also need to account for potential changes in vector species, distribution and receptivity in regions with historical malaria transmission.

The magnitude of imported malaria cases are a function of several factors including the transmission intensity in origins, the volume of travellers, the activities undertaken in the location, with some demographic groups have substantially higher infection rates [44]. For instance, malaria imported to North America and Europe is often reported in migrants returning from visiting friends and relatives in origin countries. However, the Chinese labours returning China have mainly contributed to the increasing importation of malaria into China. Lai et al. [27] have explored the driving factors of *P. falciparum* malaria importation from Africa to China, and found that the endemicity of malaria in originates and investment from China had stronger correlations with the magnitude of malaria importation, comparing to total volume of travellers. Some specific investment, e.g. resource extraction and infrastructure sectors, might have driven the huge number of Chinese workers into the regions with intensive transmission in Africa [15, 16, 45].

Moreover, the modularity analysis is of importance for understanding the structure and function of malaria importation networks [44, 46]. By mapping communities on the imported malaria network defined here, we identified groups of countries that show strong links in terms of movements of infected travellers. Certain routes from endemic countries to provinces of China carry substantially more infections than others, with evidence of tight couplings that reflect historical, culture, geographic, or economic ties [16, 27, 46], e.g. the first and second communities (Fig. 6). However, the huge groups of third and fourth communities might related to the relative homogeneity of the connections between origins and destinations within the same group. These communities of countries can serve to guide surveillance, develop mitigation strategies. For instance, health education could target to Chinese travellers, especially migration workers, to promote health protection behaviours when they travel in endemic areas.

Additionally, the decrease in the number of imported cases from Southeast Asia into China might be attributed to the decreasing endemicity of malaria in Southeast Asia in the last decade, even though there are increasing Chinese travellers from Southeast Asia into China in the last decades, with the volume of airline travellers from Southeast countries into China nearly quadrupled from 3.6 million in 2005 to 3.8 million in 2015 [47]. Therefore, China should also help the control and elimination of malaria in Africa and neighbouring countries in Asia, which in turn will reduce the importation risk of malaria into China.

To maintain malaria-free status in areas where malaria transmission has been interrupted, the main challenge of these susceptible areas is to prevent reintroduction by imported parasites. The tight coupling of locations highlights risks for secondary transmission following imported cases. For instance, the importation and autochthonous transmission of *P. vivax* have been presented in the regions along the China–Myanmar border, considering the similar climate and natural environment and vectors [48, 49]. Moreover, the importation of parasites may also introduce the drug-resistance malaria into China, and a longitudinal surveillance of drug resistance in *P. falciparum* along the China–Myanmar border revealed the persistent circulation of multidrug resistant parasites [50]. Surveillance systems need to be well planned and managed to ensure timely case detection and prompt response to prevent and interrupt onward transmission in China [49]. Regarding to the high-risk border areas in southwest China, it is vital to stick to the ‘1-3-7’ approach to promote and maintain the malaria elimination along the border [51].

There are some limitations in this study. First, the quality of data might be influenced by the key steps in public health surveillance, including availability of healthcare, the accuracy of diagnosis, accessibility of laboratory tests, and the underreporting of imported malaria due to a health worker not thinking of testing for malaria [20]. Second, it was also possible that passive data were biased toward the identification and reporting of imported (rather than local) malaria if health workers preferentially tested individuals with travel history, especially for those who travelled abroad in short-term period, but biologically their infection could not have been obtained during that time given the incubation period. Third, the places where local transmission persisted longest might be also accounted for many factors, not only the vector species and density, but also the number of imported cases, control measure and case management, urbanization, and ecological changes. However, the driving factors of malaria importation and local transmission were not quantified in this study.

## Conclusion

The results of this study illustrate the changing epidemiology of autochthonous and imported malaria in China with the significant progress made towards elimination, but the foreseeable challenges of residual autochthonous transmission and imported parasites are also highlighted. Strong surveillance and response systems need to be maintained to monitor residual transmission and introduced risk in susceptible areas. Additionally, as only a few countries in Africa and southeast Asia are expected to eliminate malaria by 2020 [1, 39], China should also take the responsibility to help the control and elimination of malaria in Africa and Southeast Asia, which in turn will consolidate the achievement of malaria elimination in China and contribute to global malaria eradication [52].

## Additional files

**Additional file 1: Table S1.** The list of variables in the individual dataset of malaria cases, 2011–2016.

**Additional file 2: Table S2.** Characteristics of *Plasmodium* malaria cases reported in mainland China, 2011–2016.

**Additional file 3: Fig. S1.** Geographic distribution of autochthonous and imported malaria by county in China, 2012–2015.

**Additional file 4: Fig. S2.** Distribution of autochthonous *Plasmodium* malaria and *Anopheles* mosquitoes in China.

**Additional file 5: Table S3.** Characteristics of *Plasmodium* malaria cases imported from Africa and Southeast Asia into mainland China, 2011–2016.

**Additional file 6: Table S4.** The community of origin–destination networks of malaria imported into mainland China, 2011–2016.

**Additional file 7: Fig. S3.** Geographic distribution of imported *Plasmodium* malaria in mainland China by origins, 2011–2016. (A) All imported cases. (B) Cases imported from Africa. (C) Cases imported from southeast Asia. (D) Cases imported from other regions. To visualize the geographic distribution of imported cases based on the location of illness onset, density maps were created and smoothed by kernel density estimation at a spatial resolution of 0.083333 decimal degrees per pixel (approx. 10 km at the equator).
