# Supplementary material for: Changing epidemiology and challenges of malaria in China towards elimination
Source: Malar J. 2019 Mar 29;18:107. doi: 10.1186/s12936-019-2736-8 (PMC6440015; doi:10.1186/s12936-019-2736-8)
Supplement: Supplementary file 1 — Additional file 1: Table S1. The list of variables in the individual dataset of malaria cases, 2011–2016. [file 12936_2019_2736_MOESM1_ESM.docx]

**Additional file 1: Table S1.** **The list of variables in the individual dataset of malaria cases, 2011-2016.**

| **Variables** | **Definition/classification** | **Completeness**  **(N = 21,062)** |
| --- | --- | --- |
| Type of diagnosis | Clinical diagnosed or laboratory-confirmed case | 100% reported |
| Type of *Plasmodium* | *P. falciparum, P. vivax, P. ovale, P. malariae,* mixed infections, or untyped | 100% reported |
| Age | The interval time from the date of birth to the date of onset | 99.96% reported |
| Nationality | Chinese or foreigner | 100% reported |
| Origin country | The last countries with malaria transmission and visited by imported cases before returning to China were recorded as the potential origins of infections. | 97.5% reported |
| Date of onset | The date of illness onset | 100% reported |
| Outcome | Fatal or not-fatal | 100% reported |
| Purpose of travel of Chinese imported cases | Labour service or other | 98.4% reported |
| Duration in other countries of Chinese cases | Days in other countries | 23.4% reported |
| Address of illness onset | The address (community/village) of case with illness onset | 100% reported |
| Coordinates of address | Latitude and longitude of address of case with illness onset | 100% produced |
| County's code of hospital | A unique 8-digital number for each county | 99.97% reported |

Note: The completeness of each variable was defined as the percentage of cases with data reported for each variable among all cases in the dataset
