# Supplementary material for: Changing epidemiology and challenges of malaria in China towards elimination
Source: Malar J. 2019 Mar 29;18:107. doi: 10.1186/s12936-019-2736-8 (PMC6440015; doi:10.1186/s12936-019-2736-8)
Supplement: Supplementary file 2 — Additional file 2: Table S2. Characteristics of Plasmodium malaria cases reported in mainland China, 2011–2016. [file 12936_2019_2736_MOESM2_ESM.docx]

## Additional file 2: Table S2. Characteristics of *Plasmodium* malaria cases reported in mainland China, 2011-2016.

| **Characteristics** | **Total (n=21,062)** | **Imported cases (n=19,154)** | **Autochthonous cases (n=1,908)** |
| --- | --- | --- | --- |
| Type of diagnosis | | | |
| Laboratory-confirmed | 19,882 (94.4%) | 18,654 (97.4%) | 1,228 (64.4%) |
| Clinically diagnosed | 1,180 (5.6%) | 500 (2.6%) | 680 (35.6%) |
| Sex |  |  |  |
| Male | 19,292 (91.6%) | 18,069 (94.3%) | 1,223 (64.1%) |
| Female | 1,770 (8.4%) | 1,085 (5.7%) | 685 (35.9%) |
| Outcome |  |  |  |
| Non-fatal | 20,924 (99.3%) | 19,017 (99.3%) | 1,907 (99.9%) |
| Fatal | 138 (0.7%) | 137 (0.7%) | 1 (0.1%) |
| Age | | | |
| Median (yrs, IQR) | 39.0 (29.2, 46.1) | 38.7 (29.4, 46.0) | 42.0 (26.1, 58.0) |
| Nationality |  |  |  |
| Chinese | 19,904 (94.5%) | 17,996 (94%) | 1,908 (100%) |
| Foreigner | 1,158 (5.5%) | 1,158 (6%) | 0 (0) |
| Species of *Plasmodium* | | | |
| *P. falciparum* | 12,006 (57%) | 11,914 (62.2%) | 92 (4.8%) |
| *P. vivax* | 7,324 (34.8%) | 5,613 (29.3%) | 1,711 (89.7%) |
| *P. ovale* | 822 (3.9%) | 821 (4.3%) | 1 (0.1%) |
| *P. malariae* | 252 (1.2%) | 248 (1.3%) | 4 (0.2%) |
| Mixed infections | 260 (1.2%) | 255 (1.3%) | 5 (0.3%) |
| Untyped | 398 (1.89%) | 303 (1.6%) | 95 (4.98%) |

Note: Data are presented as no. (%) of patients unless otherwise indicated. The autochthonous cases also included six cases of transfusion infections (3 *P. falciparum*, 2 *P. malariae*, and 1 *P. ovale*) and one suspected vertical transmission for *P. vivax.*
