# Supplementary material for: Changing epidemiology and challenges of malaria in China towards elimination
Source: Malar J. 2019 Mar 29;18:107. doi: 10.1186/s12936-019-2736-8 (PMC6440015; doi:10.1186/s12936-019-2736-8)
Supplement: Supplementary file 3 — Additional file 3: Fig. S1. Geographic distribution of autochthonous and imported malaria by county in China, 2012–2015. [file 12936_2019_2736_MOESM3_ESM.docx]

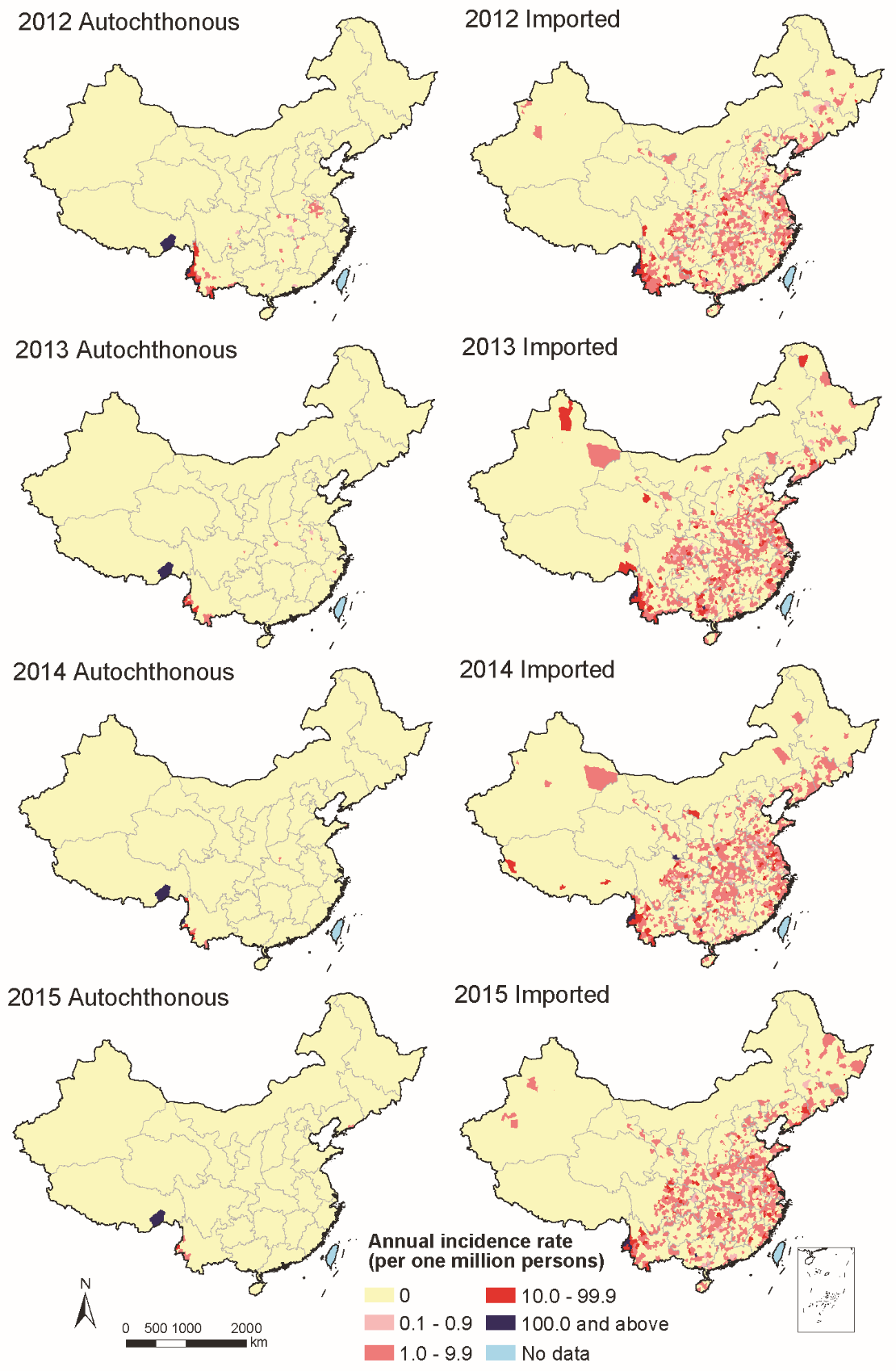


**Additional file 3: Fig. S1. Geographic distribution of autochthonous and imported malaria by county in China, 2012-2015.**
