# Supplementary material for: Changing epidemiology and challenges of malaria in China towards elimination
Source: Malar J. 2019 Mar 29;18:107. doi: 10.1186/s12936-019-2736-8 (PMC6440015; doi:10.1186/s12936-019-2736-8)
Supplement: Supplementary file 4 — Additional file 4: Fig. S2. Distribution of autochthonous Plasmodium malaria and Anopheles mosquitoes in China. [file 12936_2019_2736_MOESM4_ESM.docx]

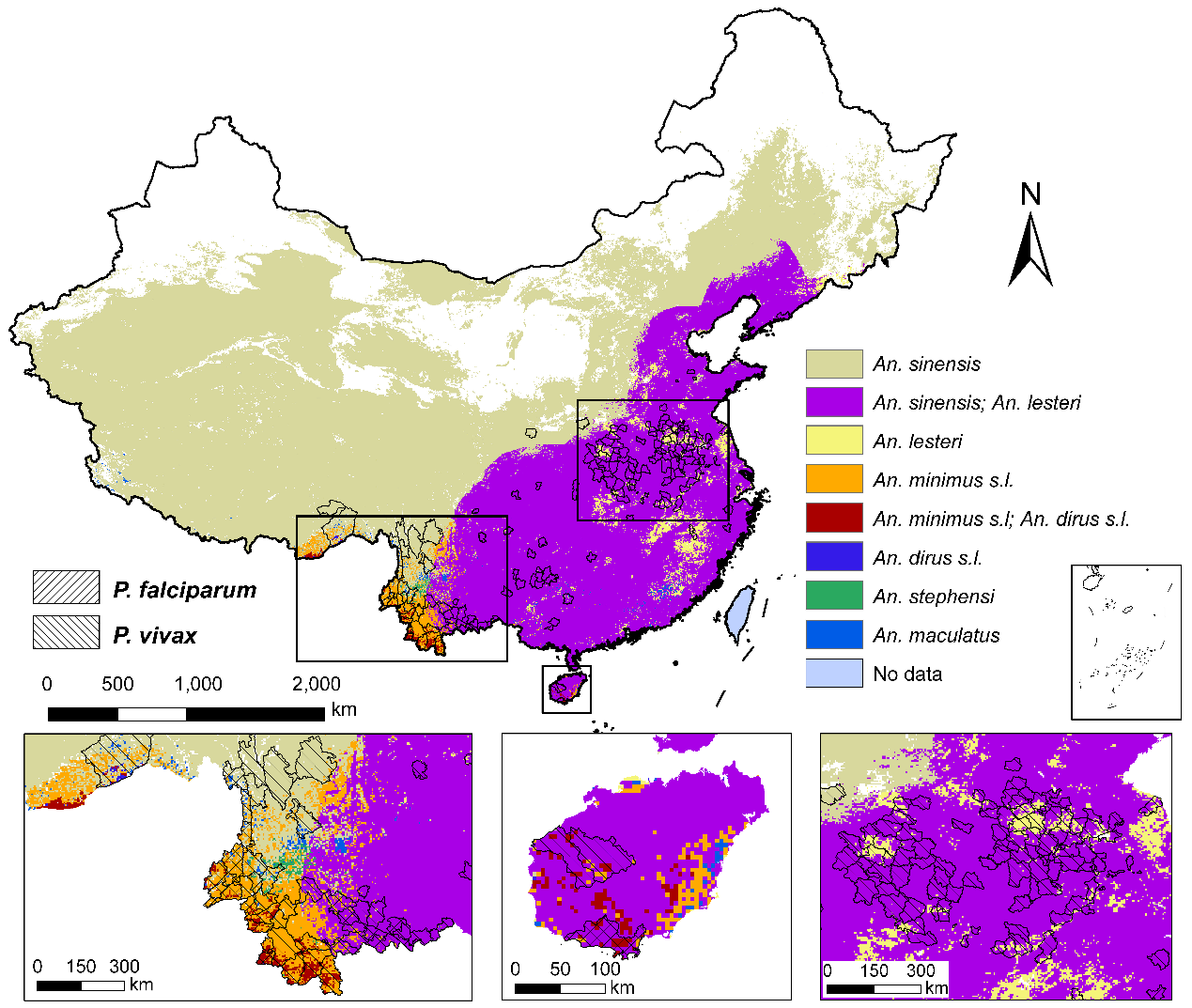


## Additional file 4: Fig. S2. Distribution of autochthonous *Plasmodium* malaria and *Anopheles* mosquitoes in China.
