# Supplementary material for: Changing epidemiology and challenges of malaria in China towards elimination
Source: Malar J. 2019 Mar 29;18:107. doi: 10.1186/s12936-019-2736-8 (PMC6440015; doi:10.1186/s12936-019-2736-8)
Supplement: Supplementary file 5 — Additional file 5: Table S3. Characteristics of Plasmodium malaria cases imported from Africa and Southeast Asia into mainland China, 2011–2016. [file 12936_2019_2736_MOESM5_ESM.docx]

**Additional file 5: Table S3.** **Characteristics of *Plasmodium* malaria cases imported from Africa and Southeast Asia into mainland China, 2011-2016.**

| **Characteristics** | **Total (n=18,519)** | **Africa (n=13,728)** | **Southeast Asia (n=4,791)** | |
| --- | --- | --- | --- | --- |
| Type of cases | | | |  |
| Laboratory-confirmed | 18132 (97.9%) | 13467 (98.1%) | 4665 (97.4%) | |
| Clinically diagnosed | 387 (2.1%) | 261 (1.9%) | 126 (2.6%) | |
| Sex |  |  |  | |
| Male | 17512 (94.6%) | 13235 (96.4%) | 4277 (89.3%) | |
| Female | 1007 (5.4%) | 493 (3.6%) | 514 (10.7%) | |
| Age | | | |  |
| Median (yrs, IQR) | 38.9 (29.6, 46.0) | 40.0 (31.0, 46.4) | 34.0 (25.7, 43.4) | |
| Nationality |  |  |  | |
| Chinese | 17477 (94.4%) | 13304 (96.9%) | 4173 (87.1%) | |
| Foreigner | 1042 (5.6%) | 424 (3.1%) | 618 (12.9%) | |
| Hospitalization | | | |  |
| Yes | 10192 (55%) | 8775 (63.9%) | 1417 (29.6%) | |
| No | 7559 (40.8%) | 4558 (33.2%) | 3001 (62.6%) | |
| Unknown | 768 (4.1%) | 395 (2.9%) | 373 (7.8%) | |
| Complications | | | |  |
| Yes | 1812 (9.8%) | 1469 (10.7%) | 343 (7.2%) | |
| No | 15420 (83.3%) | 11379 (82.9%) | 4041 (84.3%) | |
| Unknown | 1287 (6.9%) | 880 (6.4%) | 407 (8.5%) | |
| Year of onset | | | |  |
| 2011 | 2818 (15.2%) | 1407 (10.2%) | 1411 (29.5%) | |
| 2012 | 2413 (13%) | 1511 (11%) | 902 (18.8%) | |
| 2013 | 3904 (21.1%) | 3193 (23.3%) | 711 (14.8%) | |
| 2014 | 2980 (16.1%) | 2304 (16.8%) | 676 (14.1%) | |
| 2015 | 3174 (17.1%) | 2534 (18.5%) | 640 (13.4%) | |
| 2016 | 3230 (17.4%) | 2779 (20.2%) | 451 (9.4%) | |
| Month of onset | | | |  |
| January | 1601 (8.6%) | 1319 (9.6%) | 282 (5.9%) | |
| February | 1246 (6.7%) | 980 (7.1%) | 266 (5.6%) | |
| March | 1124 (6.1%) | 840 (6.1%) | 284 (5.9%) | |
| April | 1618 (8.7%) | 1071 (7.8%) | 547 (11.417%) | |
| May | 2151 (11.6%) | 1269 (9.2%) | 882 (18.4%) | |
| June | 2548 (13.8%) | 1780 (13%) | 768 (16%) | |
| July | 2008 (10.8%) | 1506 (11%) | 502 (10.5%) | |
| August | 1425 (7.7%) | 1062 (7.7%) | 363 (7.6%) | |
| September | 1254 (6.8%) | 1007 (7.3%) | 247 (5.2%) | |
| October | 1224 (6.6%) | 1010 (7.4%) | 214 (4.5%) | |
| November | 1107 (6%) | 886 (6.5%) | 221 (4.6%) | |
| December | 1213 (6.6%) | 998 (7.3%) | 215 (4.5%) | |
| Median of time delay (days, IQR) | | | |  |
| From illness onset to diagnosis | 3.6 (1.7, 6.7) | 3.5 (1.6, 6.7) | 3.7 (2.0, 6.7) | |
| From diagnosis to report | 0.2 (0.03, 0.9) | 0.3 (0.03, 1.0) | 0.2 (0.03, 0.8) | |
| From illness onset to report | 3.6 (1.7, 6.5) | 3.5 (1.7, 6.4) | 4.0 (2.5, 6.7) | |
| Median of duration abroad (days, IQR) | 307 (149, 522) | 325 (168, 547) | 117 (59, 227) | |
| Species | | | |  |
| *P. falciparum* | 11771 (63.6%) | 10874 (79.2%) | 897 (18.7%) | |
| *P. vivax* | 5257 (28.4%) | 1483 (10.8%) | 3774 (78.8%) | |
| *P. ovale* | 809 (4.368%) | 791 (5.8%) | 18 (0.4%) | |
| *P. malariae* | 247 (1.3%) | 228 (1.7%) | 19 (0.4%) | |
| Mixed infections | 253 (1.4%) | 202 (1.5%) | 51 (1.1%) | |
| Untyped | 182 (0.98%) | 150 (1.1%) | 32 (0.67%) | |
| Onset location vs report location | | | |  |
| In same county | 10831 (58.5%) | 7203 (52.5%) | 3628 (75.7%) | |
| In different counties of same province | 6743 (36.4%) | 5802 (42.3%) | 941 (19.6%) | |
| In different provinces | 945 (5.1%) | 723 (5.3%) | 222 (4.6%) | |
| Report location vs home/living location | | | |  |
| In same county | 8795 (47.5%) | 6290 (45.8%) | 2505 (52.3%) | |
| In different counties of same province | 6758 (36.5%) | 5602 (40.8%) | 1156 (24.1%) | |
| In different provinces | 1924 (10.4%) | 1412 (10.3%) | 512 (10.7%) | |
| Foreigner | 1042 (5.6%) | 424 (3.1%) | 618 (12.9%) | |
| The admin level of hospitals for diagnosis and report | | | |  |
| Province | 2628 (14.2%) | 2424 (17.7%) | 204 (4.3%) | |
| Prefecture | 5686 (30.7%) | 5088 (37.1%) | 598 (12.5%) | |
| County | 8551 (46.2%) | 5878 (42.8%) | 2673 (55.8%) | |
| Township and lower | 1653 (8.9%) | 338 (2.5%) | 1315 (27.5%) | |
| Unknown | 1 (0.01%) | 0 (0) | 1 (0.02%) | |

Note: Data are presented as no. (%) of patients unless otherwise indicated.
