# Supplementary material for: Changing epidemiology and challenges of malaria in China towards elimination
Source: Malar J. 2019 Mar 29;18:107. doi: 10.1186/s12936-019-2736-8 (PMC6440015; doi:10.1186/s12936-019-2736-8)
Supplement: Supplementary file 6 — Additional file 6: Table S4. The community of origin–destination networks of malaria imported into mainland China, 2011–2016. [file 12936_2019_2736_MOESM6_ESM.docx]

**Additional file 6: Table S4. The community of origin-destination networks of malaria imported into mainland China, 2011-2016.**

| **Community** | **Origin countries**  **(n=68)** | **Provinces in mainland China**  **(n=31)** |
| --- | --- | --- |
| 1 | Myanmar, Laos, and Thailand (3) | Yunnan (1) |
| 2 | Ghana, Cameroon, and Comoros (3) | Guangxi (1) |
| 3 | Angola, Brunei Darussalam, Burundi, Chad, Ecuador, Equatorial Guinea, Ethiopia, Gabon, Guinea, Ivory Coast, Liberia, Madagascar, Namibia, North Korea, Republic of Congo, Rwanda, Saudi Arabia, Sierra Leone, Somalia, South Africa, South Korea, Sudan, Timor-Leste, Togo, Uganda, Zambia, and Zimbabwe (27) | Tianjin, Hebei, Liaoning, Jilin, Heilongjiang, Jiangsu, Anhui, Shandong, Henan, Hubei, Chongqing, Sichuan, Guizhou, Shaanxi, and Ningxia (15) |
| 4 | Afghanistan, Bangladesh, Benin, Botswana, Burkina Faso, Cambodia, Central African Republic, Democratic Republic of the Congo, Eritrea, Gambia, Guinea Bissau, Guyana, Haiti, India, Indonesia, Kenya, Lebanon, Malawi, Malaysia, Mali, Mauritania, Mauritius, Mozambique, Niger, Nigeria, Pakistan, Papua New Guinea, Philippines, Senegal, Solomon Islands, South Sudan, Suriname, United Republic of Tanzania, Venezuela, and Vietnam (35) | Beijing, Shanxi, Inner Mongolia, Shanghai, Zhejiang, Fujian, Jiangxi, Hunan, Guangdong, Hainan, Tibet, Gansu, Qinghai, and Xinjiang (14) |
