# Supplementary material for: Changing epidemiology and challenges of malaria in China towards elimination
Source: Malar J. 2019 Mar 29;18:107. doi: 10.1186/s12936-019-2736-8 (PMC6440015; doi:10.1186/s12936-019-2736-8)
Supplement: Supplementary file 7 — Additional file 7: Fig. S3. Geographic distribution of imported Plasmodium malaria in mainland China by origins, 2011–2016. (A) All imported cases. (B) Cases imported from Africa. (C) Cases imported from southeast Asia. (D) Cases imported from other regions. To visualize the geographic distribution of imported cases based on the location of illness onset, density maps were created and smoothed by kernel density estimation at a spatial resolution of 0.083333 decimal degrees per pixel (approx. 10 km at the equator). [file 12936_2019_2736_MOESM7_ESM.docx]

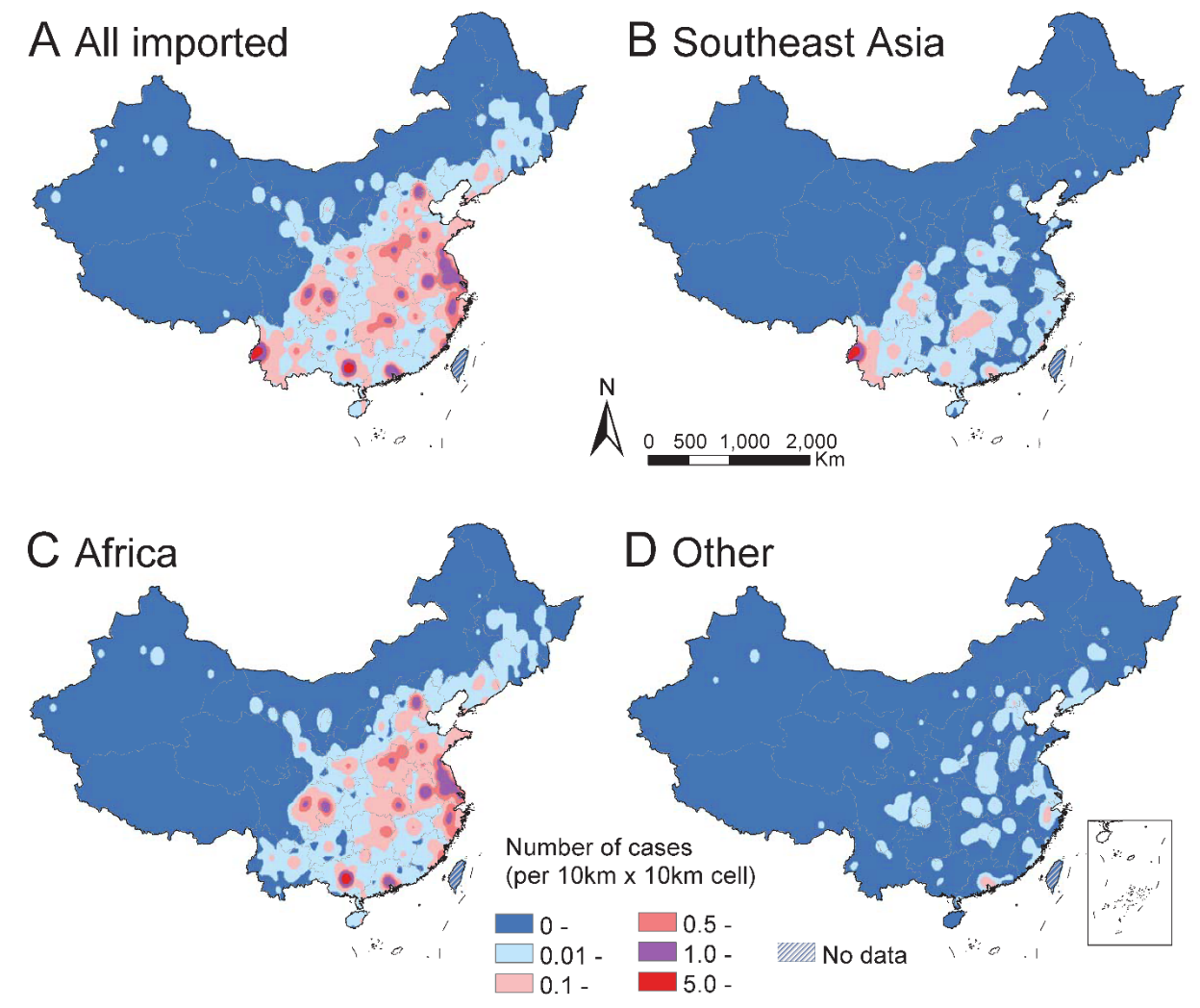


**Additional file 7: Fig. S3. Geographic distribution of imported *Plasmodium* malaria in mainland China by origins, 2011-2016.** (A) All imported cases. (B) Cases imported from Africa. (C) Cases imported from southeast Asia. (D) Cases imported from other regions. To visualize the geographic distribution of imported cases based on the location of illness onset, density maps were created and smoothed by kernel density estimation at a spatial resolution of 0.083333 decimal degrees per pixel (approx. 10km at the equator).
